# Tissue expression of MMP-9, TIMP-1, RECK, and miR338-3p in prostate gland: can it predict cancer?

**DOI:** 10.22099/mbrc.2021.40912.1646

**Published:** 2021-12

**Authors:** Rodolfo Pacheco de Moraes, Ruan Pimenta, Fernando Noboru Cabral Mori, Gabriel Arantes dos Santos, Nayara Izabel Viana, Vanessa Ribeiro Guimarães, Juliana Alves de Camargo, Katia Ramos Moreira Leite, Miguel Srougi, William Carlos Nahas, Sabrina T. Reis

**Affiliations:** 1 Laboratory of Medical Investigation (LIM55), Urology Department, Faculdade de Medicina da Universidade de São Paulo (FMUSP), São Paulo, Brazil; 2 D'Or Institute for Research and Education (IDOR), São Paulo, Brazil; 3 Instituto Do Cancer Do Estado De São Paulo (ICESP), Universidade de São Paulo, São Paulo, Brazil

**Keywords:** Image-guided biopsy, Biomarkers, Matrix metalloproteinases, TIMP proteins, MicroRNA

## Abstract

Prostate cancer is the most frequent malignancy affecting men worldwide. Due to the low sensitivity and specificity of the prostate-specific antigen test and the digital rectal exam as screening modalities, several alternatives are being studied. This study aimed to evaluate the application of MMP-9 and its regulators (TIMP-1, RECK, and miR-338-3p) as diagnostic and prognostic indicators of prostate cancer. A total of 134 randomly selected patients under investigation for prostate cancer submitted to a transrectal ultrasound-guided prostate biopsy were enrolled in the study; of these, 61 were positive for the disease (cases), and 73 were negative (control group). The tissue samples were further analyzed by gene and miR-338-3p expression analysis using qRT-PCR (one randomly selected fragment of each patient). Approximately 58% of the patients with prostate cancer presented MMP9 upregulation, while 73%, 65%, and 69% downregulated IMP-1, RECK, and miR-338-3p, respectively. MiR-338-3p was expressed at lower levels in patients with PSA concentrations exceeding 20 ng/mL (p=0.045) and abnormal DRE (p=0.006), while the RECK was more expressed in patients with abnormal DRE (p=0.01). We found that most patients with prostate cancer overexpressed MMP-9; on the other hand, most of them underexpressed TIMP-1, RECK, and miR-338-3p. MiR-338-3p presented as a possible predictor of poor prognosis. Further studies are warranted to evaluate these biomarkers as prognosis factors better.

## INTRODUCTION

Prostate cancer (PCa) is the second most common malignancy in men worldwide. In 2020, an amount of 1,414,259 new cases were diagnosed all over the world, corresponding to 7.3% of all cancers in men [1]. Many risk factors are well established: age, familial history, ethnicity, smoking, obesity, diet, and DNA repair gene mutations (e.g., BRCA1, BRCA2) [2]. Due to the low sensitivity and specificity of prostate-specific antigen (PSA) test and digital rectal exam (DRE), most frequently used as screening tests for the PCa [3-5]. Several alternatives are being studied, like magnetic resonance imaging, to increase the diagnostic of clinically significant disease and reduce the diagnostic of the clinically insignificant disease [6, 7].

Considering that cancer is a product of uncontrolled molecular pathways involved in proliferation, survival, programmed cell death, epithelium-stroma interactions, avoiding immune surveillance, among other factors, molecular biomarkers have been proposed for clinical use. During carcinogenesis, one of the leading roles performed by neoplastic cells in solid tumors is the invasion of adjacent tissues, which is possible due to the degradation of tissue barriers such as the basement membrane (BM) and extracellular matrix. A large group of proteolytic enzymes, including matrix metalloproteinases, play an essential role in this process [8, 9].

The matrix metalloproteinase type 9 (MMP-9 or gelatinase B) has been implicated in the development and progression of some types of cancer such as prostate, bladder, colorectal, and lung, due to its ability to degrade type IV collagen and gelatin, which are the main components of the BM [9, 10]. The activity of MMP-9, in turn, is regulated by endogenous inhibitors, such as tissue inhibitor of matrix metalloproteinase type 1 (TIMP-1) and reversion-inducing cysteine-rich protein with Kazal motifs (RECK). [9, 11]. 

MicroRNAs (miRNAs or miR) are small endogenous non-coding RNAs (19-25 nucleotides) that regulate gene expression by degrading mRNA or suppressing translation. The microRNA-338-3p (miR-338-3p) downregulates mRNA and protein levels of MMP-9 in liver cancer cells, suppressing cell invasion [12-14]. The study aimed to evaluate the role of MMP-9 and its regulators (TIMP-1, RECK, and miR-338-3p) for the diagnosis and prognosis of PCa.

## MATERIALS AND METHODS

**Participants:** Were enrolled 134 randomly selected patients who underwent transrectal ultrasound (TRUS) prostate biopsy at our institution between July 2015 and July 2016 for the investigation of PCa, of which 61 were diagnosed with PCa (cases) and 73 had a negative study (control group). Androgen deprivation therapy, prostate surgeries, and/or radiotherapy were the exclusion criteria for this study. Clinical, demographic, and pathological data were obtained from the institutional records.The study was approved by the local Research Ethics Committee (Faculdade de Medicina da Universidade de Sao Paulo, number 2,001,800), and all patients provided their written informed consent. 

**Variables: **We considered age, PSA level, Gleason score, and DRE results in the comparative analysis. Variables were stratified as follow: age (in years): <60, 60-70, and >70; PSA:<10ng/mL, 10-20 ng/mL, and >20ng/mL; DRE: normal, and suspect/abnormal; and, Gleason score: 6, 7, and >7. Additionally, we merge the Gleason scores 7 and >7 to compare clinically insignificant disease (Gleason score 6) to clinically significant disease (Gleason score 7).

**Genes and miR-338-3p tissue expression analysis:** One randomly selected fragment of each patient was submitted to analyze gene and miR-338-3p expressions by quantitative real-time polymerase chain reaction (qRT-PCR). We did not perform any histological analysis of the selected fragment to ensure that we selected patients with cancer, not fragments with cancer. The bench process was carried out as follows below.

 The miRVana™ miRNA Isolation Kit (Ambion, Austin, TX, USA) was used to extract RNA and miRNA from tissue samples, according to the manufacturer's recommendations. The purity and concentration of the resultant RNA were determined using a Nanodrop™ spectrophotometer (ND1000, Wilmington, DE, USA) (260/280 nM).

The miRNA cDNA was obtained using the TaqMan™ miRNA Reverse Transcription Kit (Applied Biosystems, Foster City, CA, USA). The reaction was performed using Veriti™ PCR equipment (Applied Biosystems) with the following parameters: 30 min at 16ºC, 30 min at 42ºC, and 5 min at 85ºC.

The synthesis of the cDNA of the RNA was performed using the High-Capacity cDNA Reverse Transcription™ Kit (Applied Biosystems), which uses Multiscribe™ reverse transcriptase and random primers. The solution was subjected to temperature cycles (25ºC for 10 min, 37ºC for 120 min, and 85ºC for 5 min) in the Veriti™ Thermal Cycler (Applied Biosystems). At the end of the reactions, the cDNAs were stored at -20°C until further use.

The gene and miRNA expression analysis were performed by qRT-PCR using the ABI 7500 Fast system in standard mode and TaqMan Universal Master Mix PCR (Applied Biosystems). Primers used to amplify miRNA and genes were purchased from Applied Biosystems. For endogenous controls of DNA and miRNA content, we used the b2-microglobulin gene (B2M) and the nucleolar RNA RNU48, respectively. The expression levels of all genes and miRNAs investigated were obtained through the relative quantification of expression levels determined by the 2-DDCT method.

**Statistical analysis: **The software programs STATA/SE™ 12.0 and Microsoft Excel™ 2010 were used for all statistical analyses. The Chi-Square test was used to verify the association between categorical variables. For quantitative variables, the Kolmogorov-Smirnov normality test was applied. Student’s t-test (normal distribution) and Mann-Whitney U test (non-normal) were used for comparison between two groups; ANOVA (normal distribution) and the Kruskal-Wallis (non-normal) test were used to compare more than two groups. All tests were applied with 95% confidence.

## RESULTS

**Figure 1:**
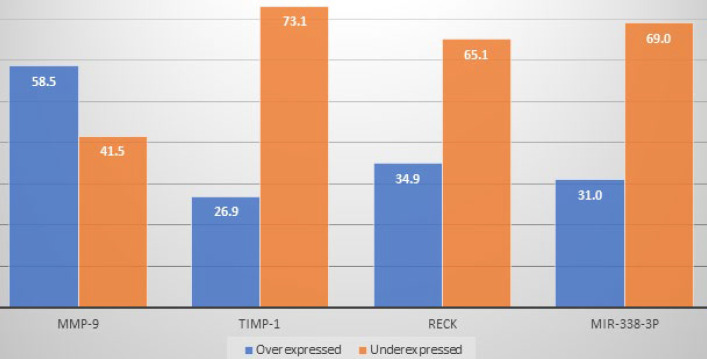
Distribution of patients with prostate cancer according to the tissue expression of the genes and miRNA

The distribution of the tests performed is summarized in Figure 1. Eight patients from the control group were excluded based on their medical records: four had a previous diagnosis of PCa and were under active surveillance; two were diagnosed for PCa upon re-biopsy, and two subjects were surgically treated for benign prostate hyperplasia (BPH) were positive for PCa after specimen analysis.

The groups of cases and controls were similar in terms of age and PSA level, with the highest proportion of patients with PCa being older than 70 years. More than 60% of the patients included in the present study had PSA levels lesser than 10ng/mL upon diagnosis, and almost half of the patients with PCa had a Gleason score of 6 (Table 1).

**Table 1:** Demographic, clinical, and pathological characteristics of the study sample

	**Prostate cancer**		
**Variables**	** Yes**	** No**	**p-value**
	** n (%)**	** n (%)**	
**Age (Years)**			
<60	13 (21.3)	12 (18.5)	0.167 *
60-70	22 (36.1)	34 (52.3)	
>70	26 (42.6)	19 (29.2)	
**PSA (ng/mL)**			
<10	40 (69.0)	39 (60.0)	0.262 *
10-20	9 (15.5)	18 (27.7)	
>20	9 (15.5)	8 (12.3)	
**Gleason score**			
6	29 (47.5)	---	---
≥7	32 (52.5)	---	
**Digital rectal exam**			
Normal	37 (75.5)	32 (78.0)	0.777 *
Abnormal/suspect	12 (24.5)	9 (22.0)	
			
	**Mean ± SD**	**Mean ± SD**	
**Age**	67.66 ± 8.73	65.69 ± 6.85	0.165 **
**PSA**	26.81 ± 106.94	10.24 ± 8.46	0.570 ***

(*) Chi-square test (**) Student’s t-test (***) Mann-Whitney U test

A higher percentage of patients with PCa had MMP-9 upregulation, while a higher rate of patients showed downregulation of TIMP-1, RECK, and miR-338-3p (Fig. 1). There was no statistically significant difference in MMP-9​​ gene expression regarding the variables evaluated in the present study (age, PSA level, Gleason score, and DRE results). The expression was higher among patients with PCa over 70 years old than patients aged 60 to 70 years and less than 60 years (p=0.16) (Table 2). A higher gene expression of TIMP-1 was found in patients with Gleason score>7 compared with those scoring 6s and 7s, but statistical significance was not reached (p=0.093). The same was observed in patients with abnormal DRE results compared with those who did not (1.05±0.65 versus 0.68±0.48; p=0.091) (Table 2).

No difference in gene expression of RECK was observed regarding age, PSA level, and Gleason score. A greater expression of this gene was seen in patients with the clinically significant disease taking into account the histological grade (Gleason scores≥7 versus 6), but statistical significance was not achieved (1.12±0.77 versus 0.71±0.69, p=0.078). In turn, patients with abnormal DRE results exhibited higher expression compared with patients with normal examination results (1.59±0.81 versus 0.84 ± 0.66, p=0.01). The miR-338-3p was shown to be less expressed in patients with a PSA level>20ng/mL compared with those with PSA of 10-20ng/mL (p=0.045). It was also less expressed in patients with abnormal DRE (0.39±0.66 versus 1.58±1.50, p=0.006). However, there was no statistically significant difference in its expression when the variables of age and Gleason score were evaluated (Table 2).

## DISCUSSION

The expression profile for MMP-9, TIMP-1, RECK, and miR-338-3p of patients diagnosed with PCa as a diagnostic tool is consistent with the hypothesis that MMP-9 plays a role in the oncogenic process that TIMP-1, RECK, and miR-338-3p act as its inhibitors.

**Table 2:** Comparison of the expression of the genes and miR-338-3p in prostate tissue samples from patients diagnosed with rrostate cancer

**Variables**	**MMP-9**	**TIMP-1**	**RECK**	**miR338-3p**
	**Mean ± SD**	**Mean ± SD**	**Mean ± SD**	**Mean ± SD**
**Age (years)**				
< 60	1.60 ± 1.82	0.63 ± 0.34	0.77 ± 0.75	1.48 ± 1.19
60-70	1.66 ± 1.60	0.75 ± 0.66	0.91 ± 0.73	1.11 ± 1.58
>70	2.76 ± 2.49	0.73 ± 0.52	0.96 ± 0.81	0.95 ± 1.14
p-value	0.160 *	0.852 *	0.876 *	0.668 **
				
**PSA (ng/mL)**				
< 10	2.02 ± 2.01	0.76 ± 0.54	0.82 ± 0.70	1.04 ± 1.13
10-20	1.41 ± 1.86	0.52 ± 0.56	0.90 ± 0.88	2.28 ± 1.92
> 20	2.92 ± 2.65	0.84 ± 0.63	1.19 ± 0.86	0.41 ± 0.66
p-value	0.404 *	0.480 *	0.491 *	0.045 **
**Gleason score**				
6	2.33 ± 1.91	0.65 ± 0.53	0.71 ± 0.69	0.93 ± 1.30
≥ 7	1.87 ± 2.29	0.77 ± 0.55	1.12 ± 0.77	1.28 ± 1.34
p-value	0.431 ¥	0.428 ¥	0.078 ¥	0.247 €
				
**DRE**				
Normal	2.11 ± 2.19	0.68 ± 0.48	0.84 ± 0.66	1.58 ± 1.50
abnormal	2.69 ± 2.29	1.05 ± 0.65	1.59 ± 0.81	0.39 ± 0.66
p-value	0.483 ¥	0.091 ¥	**0.010** ¥	**0.006** ¥

(*) ANOVA (**) Kruskal-Wallis (¥) Student t-test (€) Mann-Whitney U test

We were unable to establish any association between MMP-9 levels and the prognostic factors PSA and Gleason score. In our study, it was also not possible to observe an association between TIMP-1 gene expression and the prognostic factors “PSA” and “Gleason score” for PCa. Because this protein inhibits MMP-9, we expected its expression to be reduced in patients with worse prognoses.

Likewise, the gene expression of RECK was similar among prognostic groups for PCa. This gene was more expressed in patients with abnormal DRE findings compared with patients with normal DRE results. We expected that these patients would have lower expression levels for this gene. The effects of MMP-9 and its regulators TIMP-1 and RECK may be more associated with their relative balance rather than their isolated expression levels.

Reis et al., studied 40 prostate samples from patients with PCa who underwent radical prostatectomy (RP) using immunohistochemistry and followed the patients for an average of 92.5 months. They observed a negative relationship between TIMP-1 immunoreactivity and biochemical recurrence of the disease (22.2% of patients positive for TIMP-1 versus 56.3% of those negative for TIMP-1, p=0.042) [9].

Babichenko et al., evaluated the expression profile of MMP-9 and TIMP-1 in prostate tissue obtained from RP procedures (patients with PCa) and prostate biopsies (patients with BPH) by immunohistochemical analysis. They found that the expression of MMP-9 was lower among patients with PCa, as was the expression of TIMP-1. The authors suggest that the oncogenic effect of MMP-9 in this type of tumor is not due to its elevated expression but to decreased inhibition by TIMP-1, whose expression is significantly reduced [15].

To evaluate the expression of MMP-9, TIMP-1, and RECK in malignant prostate tissue, Reis et al. performed qRT- PCR on samples from patients diagnosed with clinically located PCa who underwent RP. They identified that MMP-9 was overexpressed in this group of patients (9.2-fold compared with controls) while TIMP-1 and RECK were underexpressed (0.75 and 0.85-fold, respectively). They also observed that MMP-9 had higher expression levels in patients with PSA>10ng/mL than patients with PSA<10ng/mL. These findings support the role of MMP-9 in the carcinogenic process [16].

In a later study, this same author demonstrated similar behavior of tissue gene expression of MMP-9, TIMP-1, and RECK in patients with urothelial carcinoma of the bladder, with most patients overexpressing MMP-9 underexpressing TIMP -1 and RECK. In this study, patients with high-grade, invasive tumors had higher MMP-9 expression levels than those with low-grade, localized disease [10].

On the other hand, in patients with colorectal carcinoma, Seubert et al., found higher expression of TIMP-1 mRNA in those who developed liver metastases than those who were free of metastases. Additionally, they performed an experimental study in rats using colorectal, pancreatic, breast, and lymphoma tumor cells. They found that elevated systemic levels of TIMP-1 created a pre-metastatic environment in the liver that diverted tumor cells to this organ but not others [17].

Rabien et al. studied RECK protein levels in prostate tissue samples after RP using Western blotting and observed that the levels of this protein were reduced by approximately 80% in tumor cells compared to the adjacent normal tissue. In addition, they demonstrated that the overexpression of RECK decreased the tissue invasion process by up to 80% in the DU-145 cell line using a marketable invasion system, although RECK overexpression did not affect cell proliferation [18].

In turn, Chen et al., reported lower RECK gene expression levels in prostate tumor cells compared with the expression in adjacent healthy cells using qRT-PCR and identified RECK as a target for miR-15b, a miRNA with oncogenic potential. Additionally, they observed that the provoked expression of RECK inhibited cell proliferation and invasive potential in PCa cell lines [19].

In our study, most patients with PCa were underexpressed for miR-338-3p. The patients with PSA > 20ng/mL and abnormal DRE findings had the lowest levels of expression. Considering that PSA and DRE are well-established markers widely used for the diagnosis and prognosis of PCa and that PSA values ​​> 20ng/mL are associated with a greater risk of metastasis, the relationship observed between miR-338-3p and these variables suggest a protective role for miR-338-3p in disease progression. However, we did not follow the same results regarding its expression when assessing the histological degree of the disease, another well-established prognostic factor.

Bakkar et al., evaluated the function of miR-338-3p in experimental models in vivo and in vitro and validated its expression in clinical samples and a cohort of localized and metastatic PCa. They observed a reduction in the expression of miR-338-3p in prostate tissue samples progressively from benign disease to localized PCa and metastatic PCa. The overexpression of miR-338-3p, in turn, dampened cell invasion and the expression of CXCL12, CXCR4, and CXCR7 chemokine signaling genes in PCa cell models in vitro [20].

The present study has some limitations. Since we did not perform additional histological analysis to determine the PCa positivity of the samples, we cannot verify any possible difference between the expression of positive and negative samples in patients with PCa. In addition, our analysis of the gene and miRNA expression patterns as prognostic factors was hampered by the small sample size of the present study.

## Conflict of Interest

The authors declare that they have no competing interests.
